# Supplementary material for: Evaluation of radiology residents’ reporting skills using large language models: an observational study
Source: Jpn J Radiol. 2025 Mar 8;43(7):1204–12. doi: 10.1007/s11604-025-01764-y (PMC12204868; doi:10.1007/s11604-025-01764-y)
Supplement: Supplementary file 1 — Supplementary file1 (PDF 214 KB) [file 11604_2025_1764_MOESM1_ESM.pdf]

Supplemental Table 1 Revised rate of first-year resident reports of CT and MRI

		First term	Last term	*P* value*
CT	Criteria 1	64%	48%	0.008
	Criteria 2	31%	20%	0.016
	Criteria 3	52%	39%	0.008
	Criteria 4	41%	34%	0.27
	Criteria 5	13%	8%	0.16
	Criteria 6	6%	7%	0.70
MRI	Criteria 1	59%	45%	0.008
	Criteria 2	28%	21%	0.22
	Criteria 3	36%	25%	0.06
	Criteria 4	41%	32%	0.06
	Criteria 5	13%	8%	0.19
	Criteria 6	4%	3%	0.75

Data showing mean percentage

*Wilcoxon Signed-Rank test

Criteria 1. Addition of missing positive findings; Criteria 2. Deletion of findings; Criteria 3. Addition of negative findings; Criteria 4. Correction of the expression of findings; Criteria 5. Correction of the interpretation of findings; Criteria 6. Proposal of additional tests or treatments

Supplemental Fig. 1

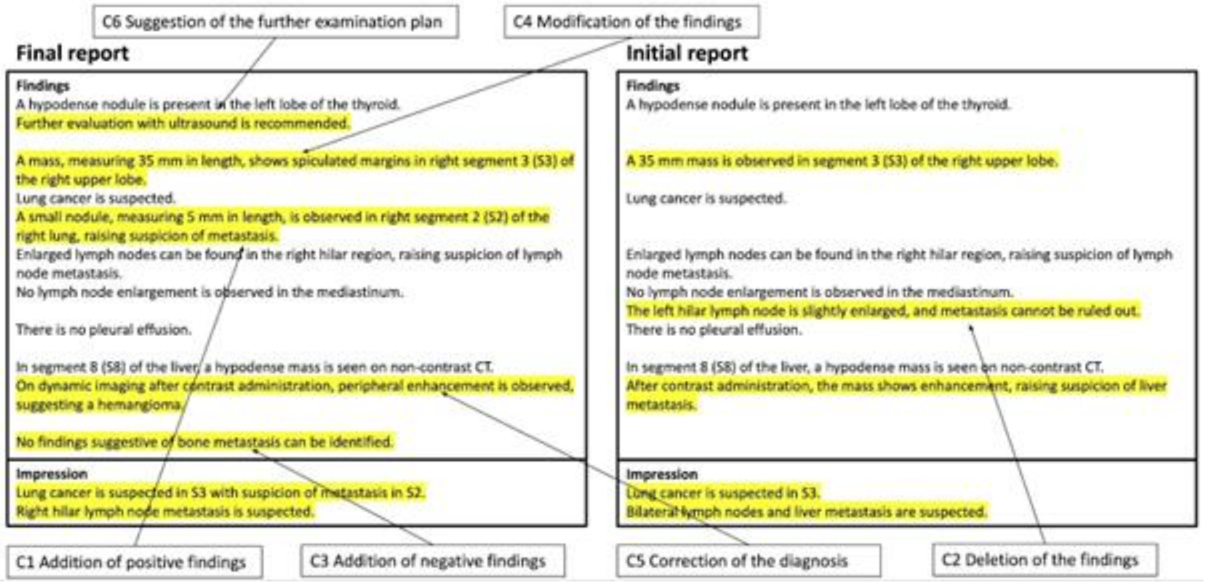

Supplemental Fig. 1 Reporting system used in this study, in which modified lines in the reports are highlighted in different colors, allowing easy visual recognition of changes. An example of criteria 1–6 is shown in C1–6

Supplemental Fig. 2

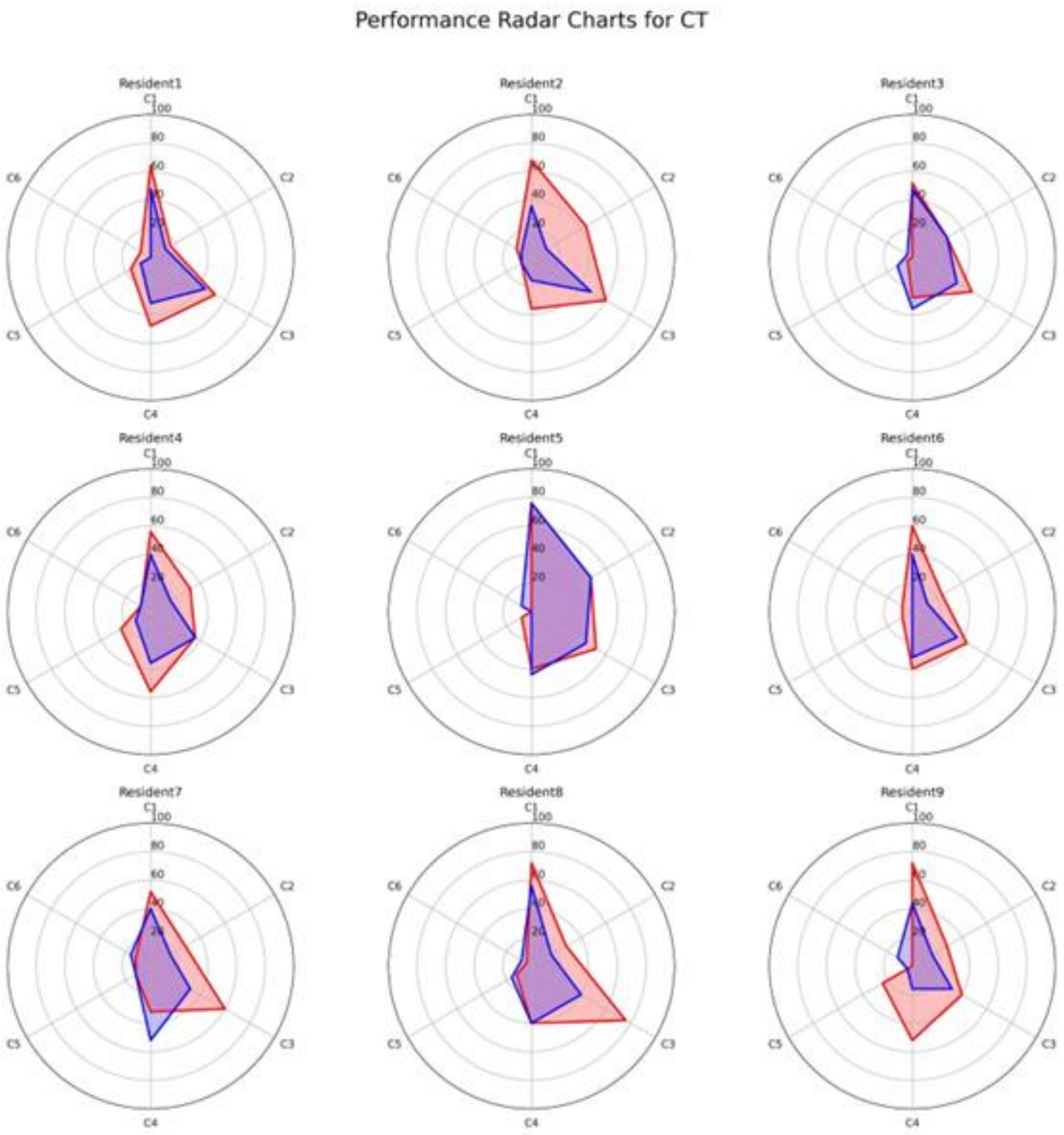

Supplemental Fig. 2 Radar chart showing the rates of CT modification for each criterion (C1–6) for nine first-year residents. Red and blue indicate the modification rates of the first and last terms, respectively. Criteria 1. Addition of missing positive findings: criterion 2. Deletion of Findings: Criterion 3. Addition of negative findings: criterion 4. Correction of the expression of findings: Criterion 5. Correction of interpretation of findings: Criterion 6. Proposals for additional tests and treatments

Supplemental Fig. 3

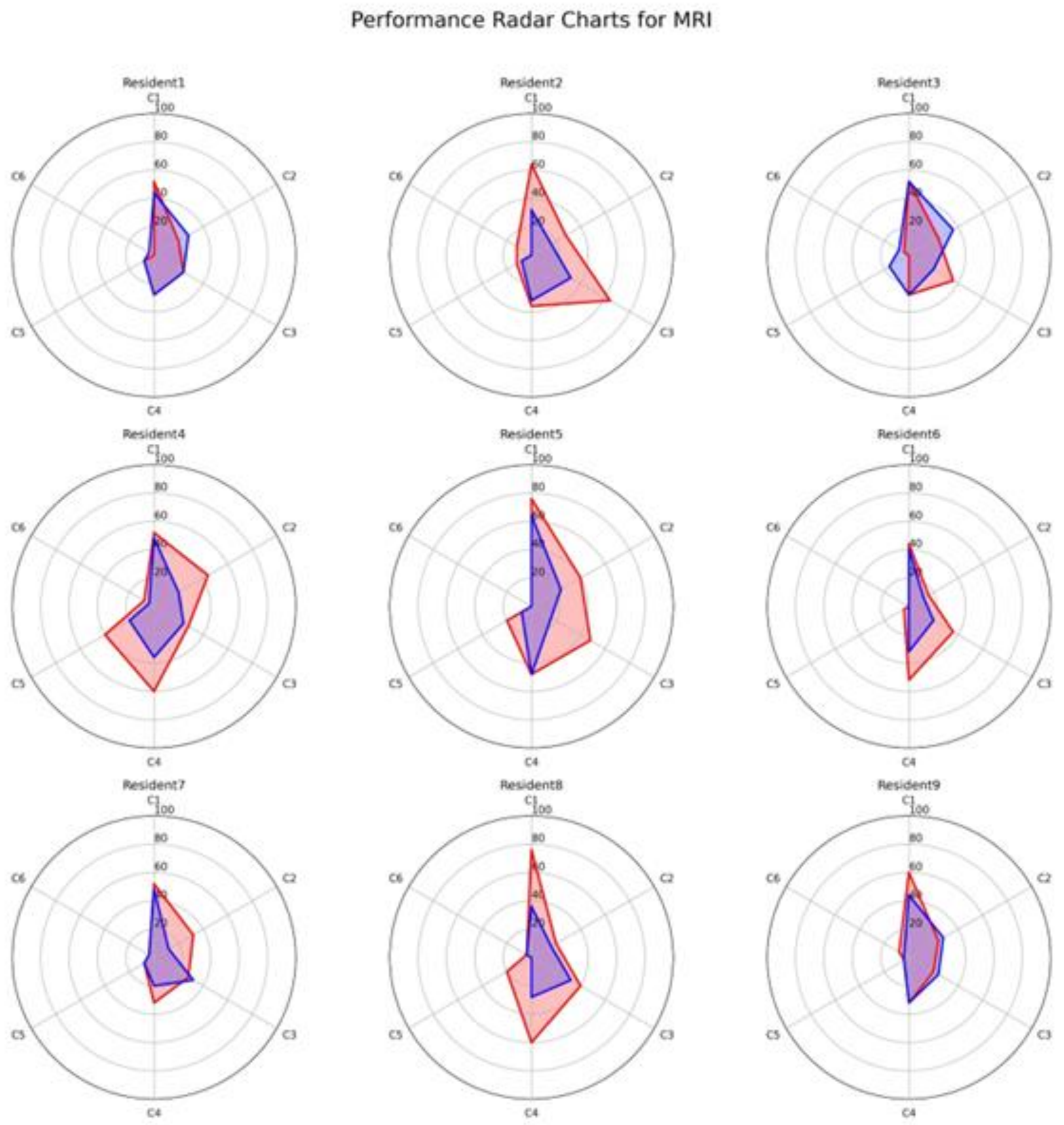

Supplemental Fig. 3 Radar chart showing the rates of MRI modification for each criterion (C1–6) for nine first-year residents. Red and blue indicate the modification rates of the first and last terms, respectively. Criteria 1. Addition of missing positive findings: criterion 2. Deletion of Findings: Criterion 3. Addition of negative findings: criterion 4. Correction of the expression of findings: Criterion 5. Correction of interpretation of findings: Criterion 6. Proposals for additional tests and treatments
